# Authors’ Reply

**DOI:** 10.1016/s2153-3539(22)00186-9

**Published:** 2011-02-26

**Authors:** Emily S. Patterson, Mike Rayo, Carolina Gill, Metin N. Gurcan

**Affiliations:** Division of Health Information Management and Systems, School of Allied Medical Professions, College of Medicine, The Ohio State University, USA; 1 Department of Industrial, Interior and Visual Communication Design, The Ohio State University, USA; 2 Department of Biomedical Informatics, The Ohio State University, USA

Sir,

We sincerely thank Professor Viroj Wiwanitkit for taking an interest in our article.[1] We agree, as we have stated in the ‘limitations’ section of the article, that the ability to generalize from our findings is limited by the small number of interviewees, particularly since they were drawn from a convenience sample. Similarly, we too would be interested in seeing the results of a study that compared the barriers and facilitators to adoption of digital images in a group with no accessibility to that in a group with accessibility.

We would like to note, however, that the use of a relatively long semi-structured interview was a deliberate methodological choice. We believed that it was important to compare what the interviewees said on similar factors, and when the factors in Table 1 were not naturally brought up by the interviewees, we questioned them directly on these points at the end of the interview. Yet, we did not structure the flow or content of the interviews based on these factors, particularly since some of these factors were only discovered as the study progressed. We made this choice because we approached our interviews with the expectation that we would have more questions than answers when we had completed our analyses. In fact, we did find this to be the case, which is why we included in Table 3 the survey questions that we plan to use in the next stage of our research. We believe that combining *exploratory approaches,* where one of the main goals is to identify the factors and variables of interest, with *confirmatory approaches*, where one of the main goals is to identify whether factors and variables generalize to a larger population, is a reasonable approach for triangulation of findings across studies.[2–4] Had we jumped directly to designing a survey or even to conducting short structured interviews, we felt that we might have missed critically important barriers and facilitators to adoption of digital images. For example, it was not until our fifth interview that we discovered that using digital images would make it easier to use barcoding so as to reduce the risk of documenting diagnostic findings for the wrong patient, and we have now included this point in our survey. Our ongoing studies are exploring different facets of this new, exciting, and rapidly developing area.

## References

[1] Patterson ES, Rayo M, Gill C, Gurcan MN (2011). Barriers and facilitators to adoption of soft copy interpretation from the user perspective: Lessons learned from filmless radiology for slideless pathology. J Pathol Inform.

[2] Potter SS, Roth EM, Woods DD, Elm WC, Chipman, Shalin, Schraagen (2000). Bootstrapping multiple converging cognitive task analysis techniques for system design. Cognitive Task Analysis.

[3] Hoffman RR (2008). Human factors contributions to knowledge elicitation. Hum Factors.

[4] Bernard HR (1940). Research methods in anthropology: Qualitative and quantitative approaches.

